# Mood Disorders Are Glial Disorders: Evidence from *In Vivo* Studies

**DOI:** 10.1155/2010/780645

**Published:** 2010-05-27

**Authors:** Matthias L. Schroeter, Hashim Abdul-Khaliq, Julia Sacher, Johann Steiner, Ingolf E. Blasig, Karsten Mueller

**Affiliations:** ^1^Department of Psychiatry, Queen Elizabeth Hospital, 10362 Berlin, Germany; ^2^Department of Molecular Cell Physiology, Institute of Molecular Pharmacology, 10125 Berlin, Germany; ^3^Day Clinic of Cognitive Neurology, University of Leipzig, 04103 Leipzig, Germany; ^4^Department of Cognitive Neurology & Nuclear Magnetic Resonance Unit, Max Planck Institute for Human Cognitive and Brain Sciences, 04103 Leipzig, Germany; ^5^Clinic for Pediatric Cardiology, University Clinic of Saarland, 66421 Homburg/Saar, Germany; ^6^Department of Psychiatry, University of Magdeburg, 39120 Magdeburg, Germany

## Abstract

It has recently been suggested that mood disorders can be characterized by glial pathology as indicated by histopathological *postmortem* findings. Here, we review studies investigating the glial marker S100B in serum of patients with mood disorders. This protein might act as a growth and differentiation factor. It is located in, and may actively be released by, astro- and oligodendrocytes. Studies consistently show that S100B is elevated in mood disorders; more strongly in major depressive than bipolar disorder. Successful antidepressive treatment reduces S100B in major depression whereas there is no evidence of treatment effects in mania. In contrast to the glial marker S100B, the neuronal marker protein neuron-specific enolase is unaltered. By indicating glial alterations without neuronal changes, serum S100B studies confirm specific glial pathology in mood disorders *in vivo*. S100B can be regarded as a potential diagnostic biomarker for mood disorders and as a biomarker for successful antidepressive treatment.

## 1. The Glial Hypothesis of Mood Disorders

Mood disorders—once considered “good prognosis diseases” —have, in fact, a less favorable outcome than previously thought [1, 2]. They are often very severe or even life-threatening illnesses. It has been suggested that impairment of neuroplasticity and cellular resilience may underlie their pathophysiology, and that optimum long-term treatment may only be achieved by the early use of agents with neurotrophic or neuroprotective effects. It has further been proposed that mood disorders are characterized by specific glial pathology [3]. Histopathological *post mortem* findings [1, 4–6] consistently showed reductions in glial cell density or glial cell numbers in prefrontal brain regions, such as the (subgenual) anterior cingulate cortex, the orbitofrontal cortex, and dorsolateral prefrontal cortex in association with reduced prefrontal gray matter in patients with mood disorders [3, 7–9]. Furthermore, alterations were described histopathologically for astrocytes [10–13] and oligodendrocytes [14–16] in these disorders. Specific reductions in oligodendrocytes have also been reported for the amygdala in major depressive disorder (MDD) [14], and microglial alterations in bipolar disorder (BD), also including manic episodes [1].

Rajkowska's hypothesis [3] of glial pathology in mood disorders has been supported by a recent study that specifically ablated astroglial cells in the prefrontal cortex of adult rats pharmacologically with L-alpha-aminoadipic acid (L-AAA) [17]. Indeed, rats treated with L-AAA showed depressive-like behavior in behavioral tests similar to depression models based on chronic unpredictable stress. Conversely, the neurotoxic ibotenate did not show any effect. Remarkably, antidepressive treatment has been shown to successfully reverse reduction in astroglial density in animal models of depression [18]. Although density and size of cortical neurons are reduced in the orbitofrontal and dorsolateral prefrontal cortices in mood disorders, these neuronal reductions seem less pronounced than glial alterations and are detected only when specific morphological size-types of neurons are analyzed in individual cortical layers [1, 6].

## 2. The Glial Marker Protein S100B in Mood Disorders

Previous studies have shown that S100B, which is found in astro- and oligodendroglia, but not in microglia in the human brain [19], is altered in both serum [20, 21] and cerebrospinal fluid in mood disorders. Cerebrospinal fluid changes have been shown for drug-free depressive patients compared with euthymic patients [22] and in animal models of mania [23]. Interestingly, the levels of the glial marker protein S100B are specifically altered in the lateral prefrontal and parietal cortices in BD [24]. Roche et al. [25] demonstrated that S100B is a susceptibility gene for BD with psychosis. Although Yang et al. [26, 27] did not find an association between S100B gene polymorphisms and MDD in a Chinese population, they revealed an influence on age of onset and subgroups (first-episode versus recurrent episode depression) of MDD.

S100 proteins are a family of acidic proteins that can bind calcium and, thus, influence various cellular responses along the calcium-signal-transduction pathway [31–34]. S100B regulates cell shape, energy metabolism, contraction, cell-to-cell communication, intracellular signal transduction, cell growth [35], and can be actively released by astro- and oligodendrocytes [19, 36]. Interestingly, the effects of extracellular S100B depend on its concentration [33, 37]. In a nanomolar concentration S100B can act as growth and/or differentiation factor for neurons and astrocytes, whereas in a micromolar concentration it may induce apoptosis. Moreover, it has been suggested that S100 proteins, such as S100B, may play a crucial role in the pathogenesis of depression and its treatment [38–44].

**Figure 1:**
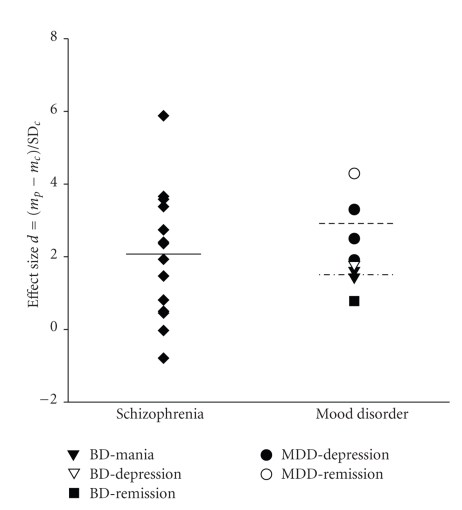
Effect sizes according to Cohen [28] of S100B serum concentration in schizophrenia, and mood disorders as identified by a systematic meta-analysis [21]. Median is shown for schizophrenia (solid line), major depressive disorder (MDD, dashed line) and bipolar disorder (BD, dashed & dotted line).

To better evaluate the relevance of S100B in mood disorders, we recently conducted a systematic, quantitative meta-analysis using MedLine and Current Contents search engines (search strategy: [S100 OR S-100] AND [depression OR mania]) [20, 21]. The following inclusion criteria were applied: diagnosis according to internationally recognized diagnostic criteria (International Classification of Diseases, ICD-10; Diagnostic and Statistical Manual of Mental Disorders, DSM-IV [45, 46]), original and peer-reviewed studies, comparison with age-matched, healthy control subjects and no overlap with cohorts of other studies. Eight studies involving 193 patients suffering from mood disorders and 132 healthy control subjects were entered into the meta-analysis. Of the patients, 86 suffered from a major depressive episode, 63 from a manic episode, and 44 were euthymic at the time of investigation. To adjust for systematic measurement effects, the effect size of each study (*d*) was calculated according to Cohen [28] as the difference of the means of the patient (*m*_*p*_) and control group (*m*_*c*_) divided by the standard deviation of the control group (*SD_c_*). This measure represents normalized elevations of S100B in the patient groups. Effect sizes of the studies are shown in Figure 1. Cohen [28] defined values of ≥0.8 as large, >0.5 as medium and >0.2 as small. The mean effect size reached high values for all episodes of mood disorders [20], namely major depressive episode of MDD (2.57 ± 0.70), manic episode of BD (1.53 ± 0.13) and currently euthymic mood disorder (2.54 ± 2.48; mean ± SD). For major depressive and manic episodes, values were significantly higher than zero, confirming high serum S100B in acute episodes of mood disorder (*T* = 6.4, = 17, *d* *f* = 2, = 1, *P* = .024, = .037; 2-tailed Student's *t*-test against 0), which was not the case for currently euthymic mood disorder (*T* = 1.4, *d* *f* = 1, *P* > .05).

We set out to compare serum S100B in BD and MDD, because these types of mood disorder are classified as separate nosological entities and because we did not find significant differences between depressive/manic episodes and remitted mood disorder per se (*P* > .05; 2-tailed unpaired Student's *t*-test) [21]. As illustrated in Figure 1, serum S100B reached high effect sizes in both MDD (3.0 ± 1.03) and BD (1.4 ± 0.44; *T* = 5.82, = 6.4, *d* *f* = 3, = 3, *P* = .01, = .008; 2-tailed one-sample Student's *t*-test against 0). Effect size was larger in MDD than BD (*T* = 2.84, *d* *f* = 6, *P* = .029; 2-tailed unpaired Student's *t*-test). For mania in BD and depression in MDD, only two studies with drug-free patients were available, each reporting high effect sizes (1.62, 3.3). Since the meta-analysis was conducted, a third study has been published with drug-free patients suffering from MDD (effect size 0,96 [47]).

Protein S100B has been detected in numerous other tissues in the human body besides glial cells, for example, in adipocytes, melanocytes, chondrocytes, myocardium, and Schwann cells [33, 35, 48]. Although changes elicited by adipocytes are at least theoretically possible in mood disorders [49, 50], no study has yet reported changes in S100B due to the aforementioned extracranial cell types.

Additionally, we compared results of the meta-analysis for mood disorders with another recent meta-analysis investigating serum S100B with the same method in 420 patients with schizophrenia [21, 51]. Although effects sizes also reached large values in schizophrenia (2.02 ± 1.78; *T* = 4.25, *d* *f* = 13, *P* = .001; 2-tailed one-sample Student's *t*-test against 0), there were no significant differences in comparison with MDD or BD (*T* = −1.03, = 0.68, *d* *f* = 16, = 16, *P* = .317, = .509; 2-tailed unpaired Student's *t*-test). In sum, results support the hypothesis that S100B is involved in the pathogenesis of mood disorders, particularly MDD.

## 3. Specificity of Elevations of Serum S100B in Mood Disorders

To validate the histopathologically generated hypothesis that mood disorders are characterized by specific glial pathology [3] *in vivo*, we recently measured S100B simultaneously with neuron-specific enolase (NSE) in the serum of patients with MDD and healthy age- and gender-matched control subjects [20]. NSE is located mainly in the cytoplasm of nerve cells and is not actively secreted [52, 53]. Hence, it has been regarded as a marker for neuronal injury or brain damage. If mood disorders are ultimately glial disorders as suggested by Rajkowska [3], one would expect elevated serum levels of S100B paralleled by unaltered neuronal marker protein NSE.

**Figure 2:**
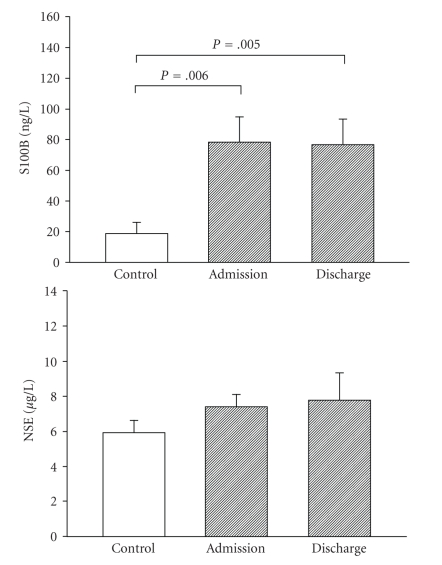
Serum concentrations of S100B and neuron-specific enolase (NSE) in patients with major depression immediately after admission and at discharge, compared with healthy age- and gender-matched control subjects [20]. *P*-values are reported for 2-tailed unpaired Student's *t*-test. Mean ± SEM.

Figure 2 illustrates serum concentration of S100B and NSE in 10 control subjects, and in 10 patients with MDD at admission and discharge. As hypothesized, S100B concentrations were higher in depressive patients at admission and discharge compared to control subjects. NSE was not statistically different between patients (at admission or discharge) and control subjects. Moreover, antidepressive treatment had no significant effect on NSE serum levels. Three other studies have reported findings on serum NSE in major depression, but the choice of the according study samples was characterized by considerable limitations. Greffe et al. [54] investigated serum NSE in 6 subjects with refractory major depression in comparison to 274 psychiatric control patients that were not characterized in more detail. Similar to our study, they found no difference between groups. Another study [55] investigated treatment effects of clinically successful electroconvulsive therapy on serum NSE in 7 patients suffering from MDD. They did not find any significant changes during treatment and concluded that values were in the normal range across all measurements without showing control data. A comparable result was reported by Agelink et al. [56] although they did not distinguish between their patients with therapy-resistant major depression and subjects with schizodepressive psychosis. These data suggest that in MDD S100B is elevated while NSE remains unaltered, providing substantial support for Rajkowska's glial hypothesis for mood disorders [3]. However, only one study [57] has investigated NSE in mania so far, showing decreased values in 30 unmedicated and 15 patients undergoing lithium treatment in comparison with 30 healthy control subjects. These results make it difficult to generalize specific glial pathology in MDD to all mood disorders and have not yet been replicated.

Increased serum levels of S100B may indicate glial alterations in mood disorders either due to brain damage [58] or due to functional secretion of S100B by astrocytes and/or oligodendrocytes [19, 36]. Mathematical models suggest that levels of serum S100B exceeding approximately 350 ng/l indicate brain damage [59]. Mean serum levels of S100B as reported in our and other studies of mood disorders do not reach this threshold [20]. Likewise, our data together with earlier studies exclude possible neuronal damage in MDD and mania as indicated by normal or even decreased serum NSE values [54, 55, 57]. One might therefore conclude that brain damage is not the primary cause of elevated S100B in mood disorders. Some authors regard serum S100B as a valid marker of blood-brain barrier integrity [59–63] and astrocytes might influence blood-brain barrier function [64–66]. Others argue that S100B might penetrate the blood-brain barrier easily, but this has not yet been proven experimentally [67, 68]. Accordingly, it remains to be clarified whether elevated serum S100B could indicate an impairment in the blood-brain barrier, as has been described for depression [69, 70].

## 4. Treatment Effects on Serum S100B

It has recently been suggested that a loss of neuroplasticity and cellular resilience may underlie the pathophysiology of mood disorders and that optimum long-term treatment can only be achieved by early neurotrophic and/or neuroprotective intervention [1, 2]. It is well-established that extracellular S100B can act as growth and/or differentiation factor for neurons and astrocytes via various intracellular signal cascades [1, 33, 71–73]. Antidepressive drugs influence the secretion of S100B by astrocytes via the serotonergic system [11, 35, 43, 44, 74–76]. S100B may even induce neurogenesis [77], which is required for the behavioral effects of antidepressants [78]. It has also been suggested that S100 proteins may play an essential role in the pathogenesis of depression and its treatment [40, 42], and that S100B-related mechanisms could be explored as potential targets for novel antidepressive therapeutics [38, 39]. Interestingly, levels of serum S100B might predict the response to antidepressive treatment in MDD [79].

To validate the impact of S100B as a marker for pharmacological treatment effects, we subsequently conducted a third systematic, quantitative meta-analysis (see above for search strategy and inclusion criteria) [20]. This meta-analysis identified three studies involving 46 patients with major depression and one study including 11 patients with mania. A fifth study examining changes in serum S100B immediately (1 and 3 hours) after electroconvulsive treatment did not include a control group [80] and because injury mechanisms following electroconvulsive treatment could represent a potential confounding factor, we did not include this study in the meta-analysis. A sixth study [56] did not differentiate between therapy-resistant major depression and schizodepressive psychosis when reporting their findings during electroconvulsive therapy, and was, accordingly, also excluded. The treatment effect size (*d*) for S100B and the severity of clinical symptoms was calculated for each study according to Cohen [28] as the difference of the means of the patient group at admission (*m*_*ad*_) and discharge (*m*_*dis*_) divided by the standard deviation at admission (*S* *D*_*ad*_). Such treatment effect size reveals a measure for relative changes from baseline.

**Figure 3:**
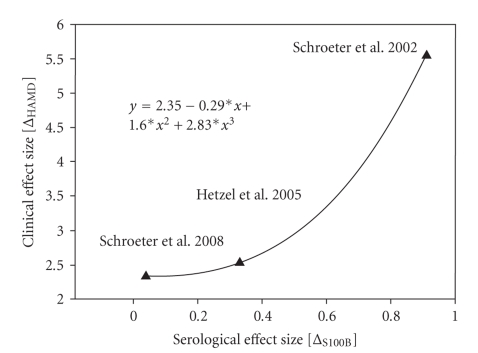
Effect sizes according to Cohen [28] for clinical (HAMD scores) and serological (serum S100B) treatment effects in major depression [20]. Effect sizes were calculated as changes between admission and discharge relative to standard deviation at admission for the three available studies [20, 29, 30]. Severity of depression was measured with the Hamilton Depression Rating Scale (HAMD).

The mean treatment effect size derived from the three available studies could be calculated for serum S100B in major depression. As expected, it reached a large value for changes on the HAMD scale (3.47 ± 1.80), with a lower impact on serum S100B (0.43 ± 0.44; *T* = 3.3, = 1.7, *d* *f* = 2, *P* = .04, = .12; 1-tailed Student's *t*-test against 0). As illustrated in Figure 3, effect sizes for clinical improvement during treatment (Hamilton Depression Rating Scale, HAMD) and respective changes of the serological marker S100B were significantly correlated with each other if the relationship for the three relevant studies involving major depression was examined (*r* = 1.0, *N* = 3, *P* < .001; correlation according to Spearman, 2-tailed *p*). This significant positive correlation between clinical treatment effects (HAMD) and serological treatment effects (S100B) indicates that serum S100B may be a reliable marker for treatment effects in major depression if clinical improvement is sufficient. For mania only one study examined changes of S100B during treatment, but without detecting any significant effects [29].

However, one has to keep in mind the limitations of the meta-analysis for these treatment studies. All of the clinical studies on serum S100B in mood disorders involved several antidepressive/antimanic drugs or combinations with other psychotropic agents such as neuroleptics when psychotic symptoms were present [20]. Likewise, treatment studies to date have experienced significant limitations of sample size. Hence, future well-powered clinical studies are necessary to overcome this limitation. Furthermore, *in vitro* (cell culture) studies examining effects of different antidepressive treatment strategies on S100B with regard to the specific signaling pathways of the neurotransmitter system mainly targeted by the antidepressant would be of high interest.

## 5. Evidence from Studies with Serum Markers for the Glial Hypothesis of Mood Disorders

To summarize findings from the literature supporting the hypothesis of glial pathology in mood disorders [3], we list the following key findings:

- Serum concentrations of the glial marker protein S100B are elevated in patients with mood disorder, major depression and mania, when compared with healthy control subjects.
- Serum S100B is higher in major depressive disorder than bipolar disorder.
- Successful antidepressive treatment reduces S100B in major depression. While only one study investigated treatment effects in mania, such an effect could not be found.
- The neuronal marker protein NSE is unaltered in major depression and its treatment. NSE is not increased in mania; the only study in the literature reported mildly reduced serum levels.
- Data support the hypothesis that elevated serum S100B is related to active secretion by astrocytes and/or oligodendrocytes in acute episodes of mood disorders, particularly major depressive disorder, and that this secretion might decline with successful antidepressive treatment.

In conclusion, these findings strongly support the concept of serum S100B as a reliable and sensitive diagnostic biomarker for mood disorders and the clinical response to antidepressive treatment in unipolar major depressive disorder. Evidence of glial changes without neuronal alterations from *in vivo* studies is consistent with the histopathologically generated hypothesis that mood disorders are characterized by specific glial pathology.
